# Long-term recovery of upper limb motor function and self-reported health: results from a multicenter observational study 1 year after discharge from rehabilitation

**DOI:** 10.1186/s42466-021-00164-7

**Published:** 2021-12-27

**Authors:** Thies Ingwersen, Silke Wolf, Gunnar Birke, Eckhard Schlemm, Christian Bartling, Gabriele Bender, Alfons Meyer, Achim Nolte, Katharina Ottes, Oliver Pade, Martin Peller, Jochen Steinmetz, Christian Gerloff, Götz Thomalla

**Affiliations:** 1 Department of Neurology, University Medical Centre Hamburg-Eppendorf, Martinistr. 52, 20246 Hamburg, Germany; 2 MediClin Klinikum Soltau, Oeninger Weg 59, 29614 Soltau, Germany; 3 RehaCentrum Hamburg GmbH, Martinistraße 66, 20246 Hamburg, Germany; 4 VAMED Klinik Geesthacht, Johannes-Ritter-Straße 100, 21502 Geesthacht, Germany; 5 Klinikum Bad Bramstedt, Klinik Für Neurologische Rehabilitation, Oskar-Alexander-Straße 26, 24576 Bad Bramstedt, Germany; 6 VAMED Rehaklinik Damp, Seute-Deern-Ring 30, 24351 Damp, Germany

**Keywords:** Stroke, Neurological rehabilitation, Recovery of function, Upper extremity, Patient outcome assessment

## Abstract

**Background:**

Impaired motor functions after stroke are common and negatively affect patients' activities of daily living and quality of life. In particular, hand motor function is essential for daily activities, but often returns slowly and incompletely after stroke. However, few data are available on the long-term dynamics of motor recovery and self-reported health status after stroke. The Interdisciplinary Platform for Rehabilitation Research and Innovative Care of Stroke Patients (IMPROVE) project aims to address this knowledge gap by studying the clinical course of recovery after inpatient rehabilitation.

**Methods:**

In this prospective observational longitudinal multicenter study, patients were included towards the end of inpatient rehabilitation after ischemic or hemorrhagic stroke. Follow-up examination was performed at three, six, and twelve months after enrollment. Motor function was assessed by the Upper Extremity Fugl-Meyer Assessment (FMA), grip and pinch strength, and the nine-hole peg test. In addition, Patient-Reported Outcomes Measurement Information System 10-Question Short Form (PROMIS-10) was included. Linear mixed effect models were fitted to analyze change over time. To study determinants of hand motor function, patients with impaired hand function at baseline were grouped into improvers and non-improvers according to hand motor function after twelve months.

**Results:**

A total of 176 patients were included in the analysis. Improvement in all motor function scores and PROMIS-10 was shown up to 1 year after inpatient rehabilitation. FMA scores improved by an estimate of 5.0 (3.7–6.4) points per year. In addition, patient-reported outcome measures increased by 2.5 (1.4–3.6) and 2.4 (1.4–3.4) per year in the physical and mental domain of PROMIS-10. In the subgroup analysis non-improvers showed to be more often female (15% vs. 55%, *p* = 0.0155) and scored lower in the Montreal Cognitive Assessment (25 [23–27] vs. 22 [20.5–24], *p* = 0.0252).

**Conclusions:**

Continuous improvement in motor function and self-reported health status is observed up to 1 year after inpatient stroke rehabilitation. Demographic and clinical parameters associated with these improvements need further investigation. These results may contribute to the further development of the post-inpatient phase of stroke rehabilitation.

*Trial registration*: The trial is registered at ClinicalTrials.gov (NCT04119479).

## Background

Stroke remains one of the greatest challenges in healthcare, as it is a common cause of acquired long-term disability in adults, and the second leading cause of death worldwide [20, 25, 42, 43]. Moreover, it will continue to be an important topic, given that demographic trends with an aging population are expected to result in a significant increase in stroke patients in the future [16]. In Germany, for example, approximately 196,000 first-time strokes occur annually, in addition to approximately 66,000 recurrent strokes [22]. On average, 25% of all patients after stroke or transient ischemic attack are discharged to an inpatient neurological rehabilitation facility immediately after acute treatment [22, 23]. Affected individuals often face dramatic changes in their daily lives, making self-reported measures of individual health status valuable for assessing long-term recovery from stroke [20, 42]. However, these measures are rarely collected, and when they are, it is in very different ways and usually not through repeated measurements, making it difficult to draw conclusions about their progression [1, 7]. Perceived health is closely related to the ability to live independently. After a stroke, however, motor impairments occur in approximately 80% of all cases and are associated with permanent disability and dependence in at least 30% [14, 30]. Therefore, much of post-stroke treatment will continue to rely on rehabilitation [29]. Well-founded evidence on long-term outcomes and rehabilitation is, however, difficult to obtain, as there is a lack of current observational studies focusing on the chronic phase of stroke [27, 39].

The significance and importance of standardized neurorehabilitation research emerges clearly in view of these backgrounds [4]. Contrary to the considerable progress and developments in acute stroke care, there are deficits in systematic and optimized stroke aftercare, as there are hardly any structured offers for cross-sectoral therapy of stroke [37]. Research on long-term neurorehabilitation is further hampered by the strict borders between the sectors of healthcare, i.e., acute clinical care, rehabilitation, and ambulatory care [2, 21]. Data and knowledge transfer often does not take place sufficiently. This is accompanied by a lack of concrete knowledge about long-term courses of stroke recovery, especially after patients have left inpatient rehabilitation [33].

The Interdisciplinary Platform for Rehabilitation Research and Innovative Care of Stroke Patients (IMPROVE), a research collaboration between a university stroke center and five rehabilitation clinics aims to contribute to closing this knowledge gap. This multicenter collaboration will examine the course of functional recovery in stroke patients, focusing on hand motor recovery and self-reported measures on health status. In this way, the long-term course of motor function recovery and self-reported health outcomes after inpatient stroke rehabilitation will be characterized. Exploratory analysis also aims to identify potential predictors of sustained improvement in motor hand function.

## Methods

### Study design and patient cohort

We performed an observational, longitudinal, multicenter study designed to characterize the course of recovery following ischemic or hemorrhagic stroke. Patients were recruited at the end of inpatient (including dayclinic) rehabilitation at five neurological rehabilitation centers in northern Germany. Patients 18 years or older with sufficient knowledge of German and with at least minimal disability (modified Rankin score ≥ 1) were considered eligible for inclusion. Exclusion criteria included pre-existing need for care or a severe psychiatric disease. Additionally, for this analysis patients with diagnosis of subarachnoid hemorrhage, craniocerebral trauma, or transient ischemic attack were excluded. A comprehensive test array was conducted at baseline (i.e., close to the end of inpatient rehabilitation) including motor and cognition tests as well as patient-reported quality of life questionnaires. These assessments were repeated at three-, six- and twelve-months follow-up at the University Medical Center Hamburg-Eppendorf. The tests were chosen to reflect the three components of the International Classification of Functioning, Disability and Health (ICF) and comprise tests regularly used in a rehabilitation setting. The full study protocol including descriptions of the test battery was published previously  [6 ].

### Assessments of motor functions and Patient Reported Outcome Measurements (PROM)

To study the course of motor function recovery following stroke, a set of assessments were carried out that cover different aspects of motor functioning. The motor function part of the upper extremity Fugl-Meyer Assessment (FMA) is a three-point scale rating (0 = cannot perform, 1 = performs partially, 2 = performs fully) that adds up to a maximum of 66 points. Joint mobility (active and passive) of the affected side is compared to the non-affected side [18].

Grip and pinch strengths of both sides were measured using a dynamometer in an upright position with the forearm resting flat on the table. The ratio of the strength of the affected and unaffected side was calculated (affected / unaffected).

The nine-hole peg test (NHPT) is a standardized measure of finger dexterity. The time it takes to remove and reassemble nine wooden pegs from a board is measured in pegs per second. The test was terminated after a maximum of 180 s, resulting in a minimum of 0.05 pegs per second. Again, the ration of affected and unaffected side is calculated [32].

In addition to these objective tests, the Patient-Reported Outcomes Measurement Information System 10-Question Short Form (PROMIS-10) was included as a subjective measurement to the set of paper-and-pencil questionnaires at all timepoints [36]. This questionnaire's main domains comprise physical and mental health. T-scores were calculated using the test’s scoring instructions. On the T-score metric, 50 is the reference population mean with a standard deviation of 10. Lower values reflect a poorer outcome.

Motor therapy (i.e. physiotherapy or ergotherapy) duration was self-reported at every follow-up in minutes per week. In the analysis, the mean weekly duration during all follow-ups was calculated.

### Statistical analysis

Statistical analysis was performed using R programming software [35]. Descriptive statistics are reported as counts and percentages or median and interquartile range (IQR) where applicable. Outcome measurements are visualized as absolute values and changes (deltas) compared to inclusion (T0).

### Linear random coefficients models

To comprehensively describe the course of motor recovery, we fitted linear random coefficients models for every motor function variable and PROMIS-10 domain using the “lme4”-package (Version 1.1–23) in R [3]. Time after inclusion in years (T0: 0 years, FU1: 0.25 years, FU2: 0.5 years and FU3: 1 year) was considered a fixed effect with a random intercept and slope (random coefficients) for every patient. *p* values were computed from t-statistics by the “broom.mixed”-package (Version 0.2.5) [8].

### Group classification and comparison

Patients were assigned into one of two groups according to their performance in the FMA hand score (part C) after twelve months—the study’s primary outcome parameter. The hand score comprises seven performance tasks and thus results in a maximum of 14 points. Patients were considered “improvers” if they improved by at least one point (FMA hand score at 12 months—FMA hand score at baseline ≥ 1) over the course of 1 year and “non-improvers” if they stagnated or worsened (FMA hand score at 12 months—FMA hand score at baseline < 1) during this period. Group assignment was carried out only if data was available at FU3 (after 12 months) and FMA hand score at baseline was < 14 points, i.e., improvement was possible.

Comparisons between groups included demographic and clinical characteristics at baseline, cardiovascular risk factors, the Montreal Cognitive Assessment (MoCA), Index for the Assessment of Health Impairments (IMET), the Patient Health Questionnaire-4 (PHQ4). Significant testing was performed using the Chi-squared test (included in the “infer” R-package, version 0.5.1 [10]) or the Mann–Whitney-Test (included in the “stats” R-package [35]) as appropriate.

## Results

**Fig. 1:**
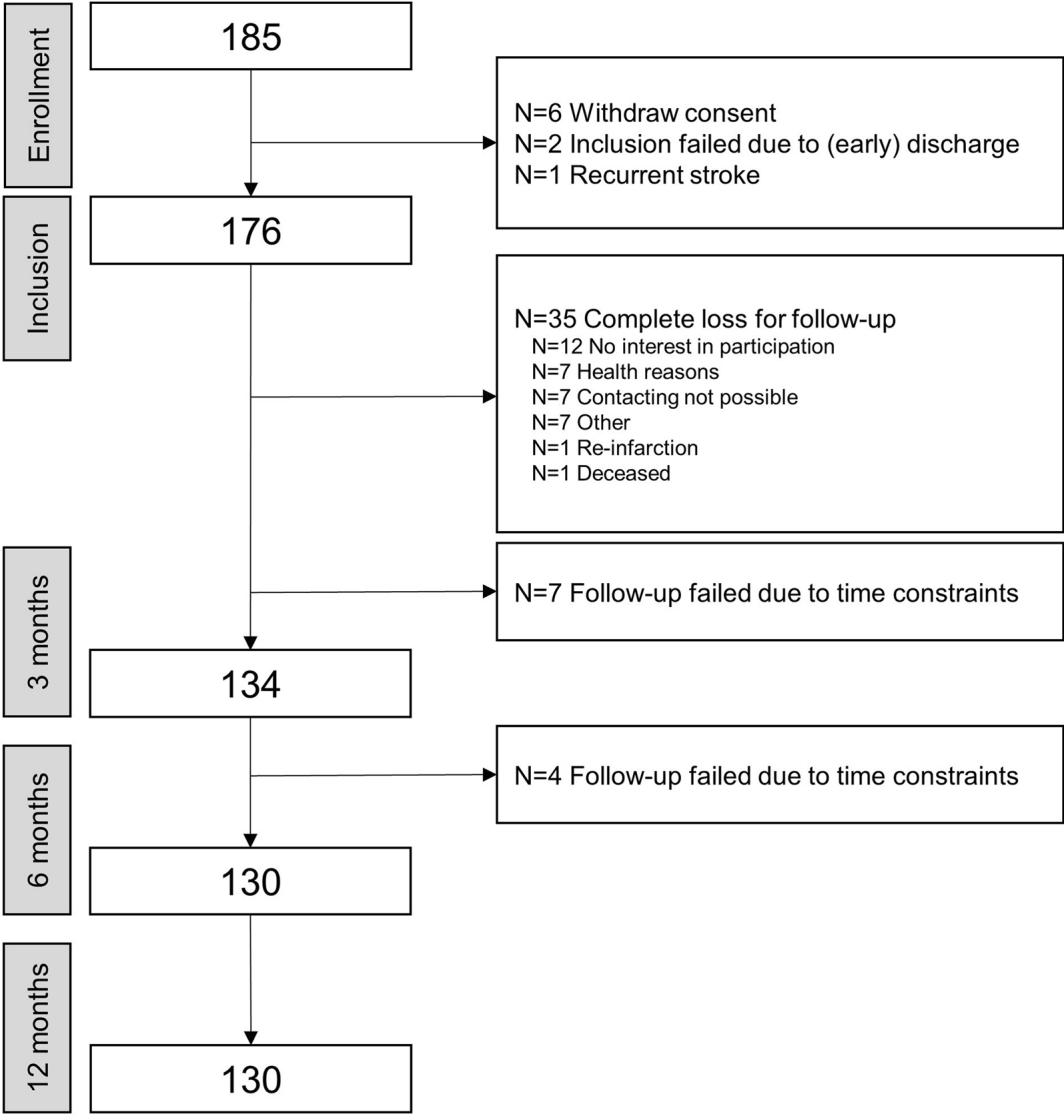
Flow-chart of participants in the IMPROVE study

Of 182 patients enrolled, 6 had to be excluded due to withdrawal of consent. Thus, 176 patients after acute stroke were included in this analysis. Patients were enrolled at the end of inpatient rehabilitation a median of 47 (37–73) days after index stroke. Follow-up at 12 months was available for 130 (73.9%) patients (see Fig. 1 for a flow chart of the study).

The median age at the time of the index stroke was 59 (52–64) years. 42 patients (23.9%) were female and 160 (90.9%) suffered from ischemic strokes. Clinical features and cardiovascular risk factors at baseline are shown in Table 1.

**Table 1:** Demographic and clinical patient characteristics at baseline

Demographic characteristics	
Female	42 (24)
Age	59 (52–64)
Stroke type	
ischemic	160 (91)
hemorrhagic	16 (9)
Clinical characteristics	
Arm paresis	118 (73)
NIHSS	2 (1–4)
mRS	1 (1–2)
Thrombolysis	32 (19)
Thrombectomy	13 (8)
Craniectomy	5 (3)
Cardiovascular risk factors	
Hypertention	140 (80)
Hyperlipidemia	112 (68)
Diabetes mellitus	31 (18)
Atrial fibrillation	15 (11)
Overweight	77 (44)
Nicotine abuse	89 (51)
Alcohol abuse	16 (9)

Values are expressed as number (percent) or median (interquartile range). NIHSS: National Institutes of Health Stroke Scale, mRS: modified Rankin Scale

**Fig. 2:**
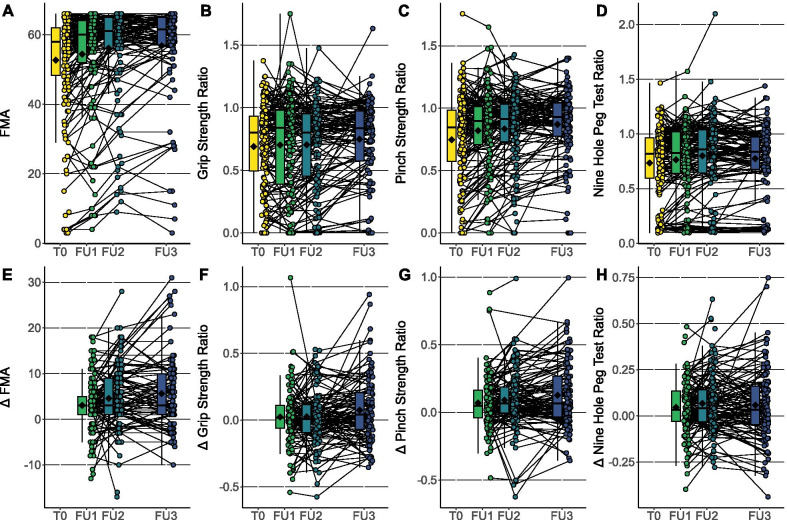
Progress of motor function recovery up to 1 year of follow up. The absolute values (upper row) and the changes (lower row) in respect to baseline (T0) are depicted for the motor function assessments FMA (**A**, **E**), grip strength ratio (**B**, **F**), pinch strength ratio (**C**, **G**) and NHPT ratio (**D**, **H**). Follow-up (FU) 1 to 3 was carried out three, six and twelve months after inclusion at the end of inpatient rehabilitation, respectively. Individual data points and box plots are reported for every follow up examination. The mean is indicated as a black rhombus

**Fig. 3:**
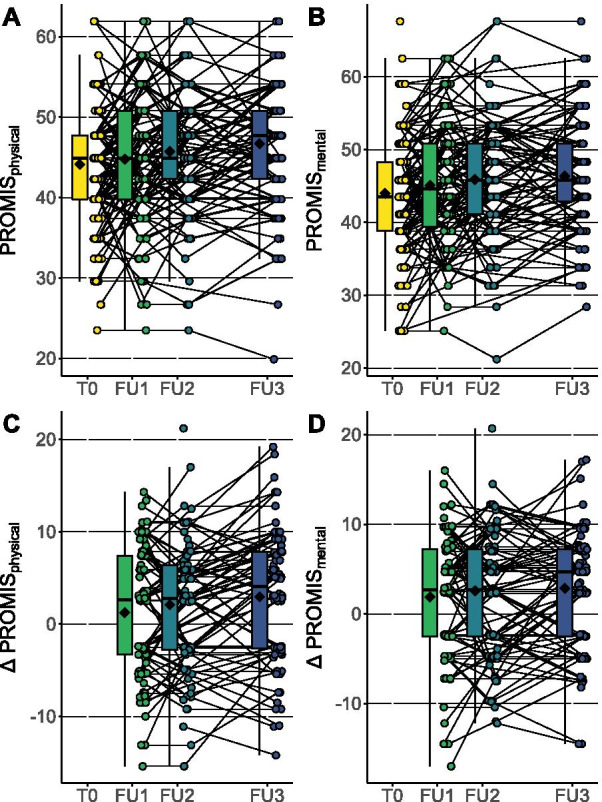
Progress of PROMIS-10 up to 1 year of follow up. The absolute values (upper row) and the changes (lower row) in respect to baseline (T0) are depicted for the physical domain (**A**, **C**) and mental domain (**B**, **D**). Follow-up (FU) 1 to 3 was carried out three, six and twelve months after inclusion at the end of inpatient rehabilitation, respectively. Individual data points and box plots are reported for every follow up examination. The mean is indicated as a black rhombus

The time course of motor function assessments and PROMIS-10 physical and mental domain is shown in Figs. 2 and 3.

Results of linear random coefficient models fitted to analyze the changes over time are presented in Table 2. The population mean of all four motor function assessments as well as of both PROMIS-10 domains improved significantly over the course of 12 months. Mean FMA score increased from 52.7 (± 15.1) to 56.8 (± 13.5) during the first year. Grip Strength Ratio and Pinch Strength Ratio were 0.69 (± 0.34) and 0.75 (± 0.36) at baseline, respectively. They increased to 0.75 (± 0.33) and 0.87 (± 0.29), respectively. NHPT increased from 0.73 (± 0.33) to 0.77 (± 0.31). The physical domain of the PROMIS-10 was 44.1 (± 7.0) at baseline, the mental domain was 44.0 (± 7.4) at baseline. Both increased to 46.7 (± 7.0) and 46.3 (± 7.2), respectively (Table 3). This increasing trend was further analyzed in linear random coefficients models. FMA scores were estimated to increase by 5.0 (3.7–6.4) points per year. Grip Strength Ratio and Pinch Strength Ratio also significantly increase by 0.07 (0.03–0.11) and 0.13 (0.09–0.17) per year, respectively. NHPT ratio was estimated to increase by 0.07 (0.04–0.1) a year. PROMIS-10 physical and mental domain showed a significant increase of 2.5 (1.4–3.6) and 2.4 (1.4–3.4) points per year, respectively.

**Table 2:** Linear random coefficients models

	Intercept	Estimate	*p* value
Motor function tests			
Fugl-Meyer Assessment	53.0 (50.7–55.3)	5.0 (3.7–6.4)	< 0.001
Grip strength ratio	0.68 (0.63–0.73)	0.07 (0.03–0.11)	< 0.001
Pinch Strength Ratio	0.75 (0.70–0.81)	0.13 (0.09–0.17)	< 0.001
Nine-Hole Peg Test Ratio	0.74 (0.69–0.79)	0.07 (0.04–0.1)	< 0.001
PROMIS-10			
Physical domain	44.2 (43.2–45.2)	2.5 (1.4–3.6)	< 0.001
Mental domain	44.3 (43.3–45.4)	2.4 (1.4–3.4)	< 0.001

Univariate models with time after inclusion in years as fixed effect. Numbers express values (95% confidence interval)

**Table 3:** Mean change of motor function tests and PROMIS-10 over 12-months follow-up

	Baseline	12-months follow-up	Delta
Motor function tests			
Fugl-Meyer Assessment	52.7 (± 15.1)	56.8 (± 13.5)	4.1
Grip Strength Ratio	0.69 (± 0.34)	0.75 (± 0.33)	0.06
Pinch Strength Ratio	0.75 (± 0.36)	0.87 (± 0.29)	0.12
Nine-Hole Peg Test Ratio	0.73 (± 0.33)	0.77 (± 0.31)	0.04
PROMIS-10			
Physical domain	44.1 (± 7.0)	46.7 (± 7.0)	2.6
Mental domain	44.0 (± 7.4)	46.3 (± 7.2)	2.3

Mean (± standard deviation) is shown for baseline and 12-months follow-up. The mean change (delta) is calculated by subtracting 12-months follow-up values from baseline values

Of 130 patients with available follow-up, 50 patients had a baseline FMA hand score < 14 and thus were available for the comparison of improvers and non-improvers. Of these, 39 (78%) patients met criteria for improvement, 11 (22%) were considered non-improvers. In the non-improver group, a higher proportion of patients was female than in the improver group (55% vs. 15%, *p* = 0.02). Non-improvers also scored significantly lower in the Montreal Cognitive Assessment (MoCA) measured at baseline (median of 22 vs. 25, *p* = 0.03). The groups did not differ in age, baseline NIHSS or mRS, risk factors or patient-reported outcomes measurements at baseline (see Table 4 for the results of group comparison).

**Table 4:** Group differences of improvers and non-improvers in the FMA hand score

	Total (n = 50)	Improver (n = 39)	Non-Improver (n = 11)	*p* value
Demographic characteristics				
Female	12 (24)	6 (15)	6 (55)	**0.0155**
Age	59 (53–67)	60 (50–65)	59 (56–74)	0.3187
Clinical characteristics				
Ischemic stroke	46 (92)	37 (95)	9 (82)	0.2069
Baseline NIHSS	4 (2–6)	4 (1.5–6)	3 (2.25–4.75)	0.6984
Baseline mRS	2 (1–2)	2 (1–2)	2 (1.5–2.5)	0.3416
Cardiovascular risk factors				
Hypertension	42 (84)	34 (87)	8 (73)	0.3533
Hyperlipidemia	37 (77)	28 (76)	9 (82)	0.7136
Diabetes mellitus	12 (24)	10 (26)	2 (18)	0.7351
Atrial fibrillation	2 (5)	2 (7)	0 (0)	1
Overweight	20 (40)	16 (41)	4 (36)	1
Nicotine abuse	21 (43)	17 (44)	4 (40)	1
Alcohol abuse	5 (10)	4 (10)	1 (9)	1
MoCA (at inclusion)	25 (22–27)	25 (23–27)	22 (20.5–24)	**0.0252**
IMET (at inclusion)	35 (24.75–52)	31 (22.5–51)	50 (35–52)	0.1805
PHQ4 (at inclusion)	3 (1–4)	2.5 (0.25–4)	3 (2–4)	0.6185
Motor therapy duration	46.7 (9–100.4)	48.3 (17.5–101.3)	26.7 (1.7–74.4)	0.3204
PROMIS-10 (at inclusion)				
Physical domain	42.3 (39.8–47.7)	42.3 (39.8–47.7)	42.3 (38.6–44.9)	0.6190
Mental domain	43.5 (41.1–45.8)	43.5 (41.1–47.68)	41.1 (38.8–44.65)	0.1446

NIHSS: National Institutes of Health Stroke Scale, mRS: modified Rankin Scale, MoCA: Montreal Cognitive Assessment, IMET: Index for the Assessment of Health Impairments, PHQ4: Patient Health Questionnaire-4, Motor therapy duration: Mean weekly minutes of physio- or ergotherapy across all follow-ups. Values are reported as numbers (percent) or median (interquartile range) where applicable. Significant *p* values are displayed in bold

## Discussion

This prospective observational multicenter study investigated the functional recovery of stroke patients within 1 year after discharge from inpatient neurorehabilitation. The data obtained, collected in a structured manner using standardized repeated measures, provide novel insights into the long-term course of recovery after stroke rehabilitation. The focus was on recovery of hand motor function and patient-reported outcome measures. In a large cohort of patients with ischemic or hemorrhagic stroke, we observed further improvement of all assessments of motor function and self-reported health state by 12 months after the end of inpatient rehabilitation. Continuous improvement was observed for both scores from external assessment as well as for self-reported outcome.

Continued recovery and adaptation to the disease are motivating for patients, researchers, and society, which is particularly important in a common chronic disease such as stroke [15]. The IMPROVE results of further improvement in hand function after completion of neurorehabilitation are consistent with previous studies, partly involving smaller sample sizes [17, 26]. At the same time, they contrast with larger studies conducted across Europe that have shown long-term deterioration in motor function and even decline within 5 years to levels similar to those seen two months post stroke [33]. Comparable results have also been reported for 3 years after stroke [31]. This suggests that there may be a turning point in the long-term course after stroke recovery where further improvement turns into functional deterioration. More likely, however, is that the selection of study participants as well as methods of analysis together with presentation of results explain these disparate findings. For example, Borschmann and Hayward [9] pointed out that recovery trajectories differ depending on whether they are considered at the individual or group level. Our results add to the recent debate about the proportional recovery rule of recovery from stroke. This rule states that approximately 70% of initial function is regained by a majority of stroke patients [24, 34]. Due to the study design, interpretation of IMPROVE within the framework of the proportional recovery rule is limited, because behavioral scores for the initial impairment were not collected and the time span between index stroke and inclusion in the trial varied from patient to patient with a median of about one and a half month. Nonetheless, our finding of continued recovery beyond six months represents an interesting addition to the 70%-theory. Further research is needed to comprehensively study the characteristics and differences of improvers and non-improvers. Knowledge of the potential for continued functional recovery offers opportunities for further, more patient-centered approaches in long-term rehabilitation. Especially since there is a substantial evidence base for rehabilitation interventions for chronic stroke [38].

The IMPROVE data provide an insight and overview into the transition between sub-acute and chronic phases of mildly affected stroke patients. In addition, the data provide a good basis for hypothesis generation. In this work, one focus was laid on a subgroup of patients with impaired hand function at inclusion. However, patients were also included even if they no longer had a deficit in hand function at baseline. This may be considered a limitation of data collection. Yet, the majority of study participants (73%) met this criterion. Another weakness of the data collection is that there was no standardized count of screened potential participants. This was not possible for organizational reasons.

Nevertheless, interesting findings can be gleaned from the data. For example, it is noticeable that in the group of improvers the percentage of women is lower than in the group of non-improvers. These gender differences are a growing focus of health research. It is now clear that, in addition to the fact that women are usually older and have different risk factors than men at the time of stroke onset, they are more impaired and have a poorer quality of life in the post-stroke course [11]. It is particularly interesting to note this difference in light of the fact that there seems to be no gender discrepancy in acute care, yet women have worse functional outcomes at three months [12]. Although the IMPROVE study was not designed to examine sex differences within long-term outcomes after stroke, our results may contribute to the necessary discussion and underpin prior research stating worse functional outcomes, poorer quality of life, and greater handicaps in women after stroke [19].

Another striking difference is the significant lower scoring in the MoCA. Along with other widely used screening assessment for detecting cognitive impairment, the MoCA has been suggested as an independent predictive variable of functional outcome after stroke [13, 40, 44]. Routine cognitive assessment may contribute to single out patients in need of closer post-inpatient rehabilitation care.

In this cohort, we observed a noteworthy decrease in the duration of outpatient motor therapy in the non-improver group. While not being statistically significant in this small patient subgroup, this finding might provide the basis of further hypothesis formation. The IMPROVE study aims primarily to illustrate and describe the long-term processes within 1 year after stroke. The interpretation of causal relationships and differences between improvers and non-improvers can only be touched upon with the present data set. Contributing to this limitation is the lack of structured imaging data collection here. This should be considered in future projects.

The first days, weeks, and months after stroke are crucial for the further development of patients, as most of the recovery takes place during this time and this period is considered to be the time window of increased neuronal plasticity [5, 28, 41]. This period after stroke is therefore often the subject of scientific research and relatively well explored. However, this does not yet apply to the long-term course, especially of motor parameters. The later stages of recovery pattern are difficult to represent [33]. IMPROVE attempts to represent these stages up to 1 year after discharge from the rehabilitation clinic and provide data for this period.

The IMPROVE project sheds light on the transition of patients from acute to post-acute stroke rehabilitation. More scientific attention should be paid to this phase, as patients are confronted with many difficult situations and decisions during this transition and the organization of this transition has been insufficient so far [21, 37]. The IMPROVE results can provide guidance for this transition, such as which patient characteristics to pay particular attention to here.

## Conclusions

One year after discharge from inpatient rehabilitation for stroke, patients have further improved their motor function and self-reported health status. Female patients, and patients with lower cognitive function were less likely to show improvement in hand function. Future studies are needed to characterize the trajectories and predictors of continuous recovery of function more precisely and to shed light on differences, also at the individual level. These results may help improve measures to support continuous further of motor function after inpatient rehabilitation.

## Data Availability

The raw data underlying this article are intended for publication on a suitable platform and can be made available by the corresponding author on reasonable request.

## Abbreviations

FMA: Fugl-Meyer Assessment
ICF: International Classification of Functioning, Disability and Health
IMPROVE: Interdisciplinary Platform for Rehabilitation Research and Innovative Care of Stroke Patients
IQR: Interquartile range
MoCA: Montreal Cognitive Assessment
mRS: Modified Rankin score
NHPT: Nine-Hole Peg Test
NIHSS: National Institutes of Health Stroke Scale
PROM: Patient Reported Outcome Measurements
PROMIS-10: Patient-Reported Outcomes Measurement Information System 10-Question Short Form
UKE: University Medical Center Hamburg-Eppendorf
